# Psychometric Properties of the QuickDASH in Patients with Neck Pain

**DOI:** 10.3390/jcm14041266

**Published:** 2025-02-14

**Authors:** Yi-Jing Lue, Kuang-I Cheng, Chih-Lung Lin, Chung-Hwan Chen, Yen-Mou Lu

**Affiliations:** 1 Department of Physical Therapy, College of Health Sciences, Kaohsiung Medical University, Kaohsiung 807, Taiwan; yijilu@kmu.edu.tw; 2 Department of Medical Research, Kaohsiung Medical University Hospital, Kaohsiung Medical University, Kaohsiung 807, Taiwan; 3 Department of Anesthesiology, Kaohsiung Medical University Hospital, Kaohsiung 807, Taiwan; kuaich@kmu.edu.tw; 4 Department of Neurosurgery, Kaohsiung Medical University Hospital, Kaohsiung 807, Taiwan; chihlung1@yahoo.com; 5 Graduate Institute of Medicine, College of Medicine, Kaohsiung Medical University, Kaohsiung 807, Taiwan; 6 Department of Orthopaedics, School of Medicine, College of Medicine, Kaohsiung Medical University, Kaohsiung 807, Taiwan; hwan@kmu.edu.tw; 7 Department of Orthopaedics, Kaohsiung Medical University Hospital, Kaohsiung 807, Taiwan

**Keywords:** neck pain, QuickDASH, reliability, validity, EFA, CFA, responsiveness

## Abstract

**Background/Objectives**: Many patients with neck pain have upper limb disorders, and prolonged use of computers at work commonly induces neck/shoulder pain. The purpose of this study was to investigate the psychometric properties of the Quick Disability of the Arm, Shoulder, and Hand (QuickDASH) in patients with neck pain. **Methods**: A total of 189 patients with neck pain were included in acceptability, reliability, validity, and responsiveness studies. The floor and ceiling effects were used for assessing acceptability. The internal reliability and test–retest reliability were used for assessing reliabilities. The construct validities (convergent/divergent validity and exploratory/confirmatory factor analyses) were used for assessing validity. The effect size (ES) and standardized response mean (SRM) were used for assessing responsiveness. **Results**: The QuickDASH had a slight floor effect (16.4%). For reliability, the internal consistency (Cα= 0.945) and test–retest reliability (ICC = 0.98; SEM = 3.17, and MDC = 8.79) were excellent. For validity, the convergent and divergent validities were satisfactory. Exploratory factor analysis revealed two factors of the QuickDASH (function factor and symptom factor), and confirmatory factor analysis confirmed the two-factor model. Responsiveness was supported by the high ES (0.85) and SRM (0.82). **Conclusions**: The QuickDASH is a reliable, valid, and responsive instrument for assessing upper limb disorders in patients with neck pain.

## 1. Introduction

Neck pain prevalence and disability have increased significantly worldwide, with more than 300 million people having neck pain lasting over 90 days [1]. Patients with neck pain commonly report upper limb symptoms. About 80% of patients report aggravation of their neck pain by upper limb activities, and higher neck pain severity is associated with greater upper limb functional restriction [2,3]. Prolonged, extensive use of computers in offices can induce musculoskeletal pain, the majority of which (58–62%) is neck/shoulder pain [4,5]. A systematic review with meta-analysis studied sedentary behavior and found that sitting in the workplace was associated with more neck/shoulder pain (OR = 1.73), as was computer usage time [6]. During the COVID-19 pandemic, millions of people worked from home, and a study of 4112 homeworkers reported that only 28.1% were free of neck/shoulder pain; most participants reported such pain, which in many cases even interfered with their work [7].

The Neck Disability Index (NDI) is commonly used for patients with neck pain. The NDI has demonstrated overall good psychometric properties [8]; however, the items do not address upper limb pain and disability. In 2022, Wiitavaara and Florin conducted a systematic review of the content and psychometric properties of measures of physical function in patients with arm–shoulder–hand disorders [9]. They identified a total of 9 questionnaires from 23 studies. The most often used questionnaires with adequate psychometric results were found to be the Disability of the Arm, Shoulder, and Hand (DASH) Outcome Measure, the QuickDASH, and the Shoulder Pain and Disability Index. However, for patients with neck/upper limb disorders, only a few research reports used the QuickDASH to examine neck/upper limb disorders [10,11].

The DASH was produced by the American Academy of Orthopaedic Surgeons (AAOS) and the Institute for Work and Health. Subsequently, the QuickDASH was developed from the DASH by Beaton et al. through three item-reduction approach methods: concept retention, equidiscriminative item–total correlation, and Rasch analysis [12]. The QuickDASH is a more efficient version, having only 11 items, and it is a reliable and valid measure of physical function and symptoms in the upper limbs. Although studies of the measurement properties of the QuickDASH have supported its good performance, with strong positive evidence for reliability and moderate to strong validities [13], research validating the QuickDASH for use in patients with neck pain has been insufficient to date.

Assessment of psychometric properties is needed for a good instrument. Psychometric properties are categorized into three types: reliability, validity, and responsiveness. Reliability concerns the stability or reproducibility of an instrument, validity indicates its accuracy, and responsiveness refers to its ability to detect changes over time in the latent trait being assessed [14]. The COSMIN (consensus-based standards for the selection of health status measurement instruments) provides the guidelines to evaluate existing patient-reported outcome measures [15]. Recently, it has become common for systemic review studies to follow these guidelines in assessing the criteria for good reliability, construct validity, and responsiveness [16]. The purpose of this study was to investigate the psychometric properties of the QuickDASH in patients with neck pain.

## 2. Materials and Methods

### 2.1. Participants

The study was a cross-sectional study. Patients with neck pain, regardless of the presence or absence of arm pain, were recruited from the pain clinic and the orthopedic and neurosurgery departments at Kaohsiung Medical University Hospital in Taiwan. They were identified by physicians based on their symptoms, physical signs, and imaging study results. The inclusion criteria were neck pain associated with diagnoses of herniated intervertebral discs, degenerative joint disease, strains and sprains, and non-specific causes. The exclusion criteria were neck pain attributed to shoulder disease, inflammatory rheumatic disease, or cancer. The study received approval from the hospital’s institutional review board (KMUH-IRB-980589 and KMUH-IRB-E(I)-20210363), and all participants provided informed consent in written form.

### 2.2. Procedure

Each participant was asked to complete questionnaires compiled in a booklet, including the Taiwan version of the QuickDASH, the Taiwanese version of the NDI, the Medical Outcomes Survey Short Form 36 (SF-36), and two types of Visual Analog Scales (VASs), namely, the VAS-N (assessing neck and shoulder pain) and the VAS-A (assessing arm and hand pain) for determination of the convergent validity. For the assessment of the test–retest reliability, patients with chronic neck pain for a minimum of 3 months and no recent changes in pain or function were recruited for the study. The retest was conducted at intervals of 7–14 days. For the responsiveness study, the participants were followed for at least 3 months (ranging from 3 to 6 months). The participants were asked to report changes in their symptoms (i.e., significant improvement or slight improvement/no change/slight deterioration or significant deterioration).

### 2.3. Instruments

#### 2.3.1. QuickDASH

The QuickDASH, which contains 11 items, was developed from the DASH through three item-reduction approaches by Beaton et al. and the Upper Extremity Collaborative Group [12]. The 11 items ask about the following: opening a jar (Q1); performing household chores (Q2); carrying a shopping bag (Q3); washing one’s back (Q4); using a knife (Q5); recreational activities with force or impacts (Q6); interference with social activities (Q7); limitations in work or daily activities (Q8); shoulder, arm, or hand pain (Q9); tingling (Q10); and difficulty sleeping (Q11). The two optional modules (work and sports/performing arts) were not used in this study. Each item is scored on a 5-point scale of 1 to 5, with the lowest numeral indicating no limitation and the highest, maximal limitation. Scores are calculated only for responses with at least 10 of the 11 items completed and transformed to a scale of 0–100, with higher scores indicating greater disability. The Taiwanese version of the DASH is a well-developed and validated questionnaire [17].

#### 2.3.2. Neck Disability Index

The NDI contains 10 items developed by Vernon and Mior. The questions were designed to understand how neck pain affects the management of activities in everyday life [8]. The 10 items cover pain intensity, the level of disability in personal care, lifting, work, headache, concentration, sleeping, driving, reading, and recreation. Each item is scored on a 6-point scale, with 0 representing no limitation and 5 representing maximal limitation. The scores of the 10 items are summed to obtain the NDI score (0–50 points). The Taiwanese version of the NDI is a well-developed and validated questionnaire [18].

#### 2.3.3. Medical Outcomes Survey Short Form 36

The SF-36 comprises eight subscales of health-related quality of life. These subscales cover physical aspects including physical functioning (PF), role limitations caused by problems with physical health (RP), bodily pain (BP), general health (GH), and vitality (VT), as well as social and mental aspects including social functioning (SF), role limitations due to emotional problems (RE), and mental health (MH) [19]. The subscale scores are transformed to a scale of 0 to 100, and higher scores indicate a better quality of life. Two summary measures, the physical component summary (PCS) and the mental component summary (MCS), are calculated from the eight health subscales, and higher scores (0–100) indicate better physical and mental function, respectively. The Taiwanese version of the SF-36 has demonstrated good reliability and validity [20].

#### 2.3.4. Data Management and Statistical Analyses

The α level was set at 0.05. Statistical analyses were conducted in IBM SPSS Statistics version 21, and the confirmatory factor analysis (CFA), in IBM SPSS AMOS version 21 (IBM Corp., Armonk, NY, USA).

##### Acceptability

To assess the acceptability of the QuickDASH, we analyzed any missing data, the observed score as compared with the possible score range, as well as possible floor and ceiling effects. For missing data, less than 5% was considered acceptable. Floor and ceiling effects exceeding 15% were considered significant. The acceptable skewness values ranged from −1 to 1 [21].

##### Reliability

1. Internal consistency

The internal consistency of the 11 items was assessed with Cronbach’s α. The corrected item–total correlations and Cronbach’s α with each item deleted were assessed. A Cronbach’s α ≥ 0.7 is acceptable [22]. Correlations for item–total correlation should be moderate to high. For each item deletion, an increase in Cronbach’s α of more than 0.1 indicates poor correlation with the scale [21].

2. Test–retest reliability

For assessment of the test–retest reliability, both the relative and absolute reliability were examined. The relative reliability was estimated with intraclass correlation coefficients (ICCs), with ICC values ≥ 0.90 indicating excellent reliability [23]. The absolute reliability was estimated with the standard error of measurement (SEM) and minimal detectable change (MDC) [24]. SEM was used to evaluate the measurement error with the formula (standard deviation of all test scores) X√ (1 − ICC).

The MDC is generally applied as the threshold indicating whether the changing scores of individual subjects are real and outside the range of the measurement error for an individual at a confidence level of 0.95, and it is calculated as 1.96 X SEM X√2. The SEM and MDC values of a good outcome measure should be low and sensitive to change related to clinical intervention.

##### Construct Validity

1. Convergent and divergent validity

The convergent validity of the QuickDASH was assessed with the correlations between the QuickDASH and the NDI, the VASs (VAS-N and VAS-A), and the SF-36 (PCS/MCS and 8 subscales). Spearman rank correlation was used to examine the correlations. We hypothesized that the QuickDASH and the NDI would have a strong correlation, and that the correlation of the QuickDASH would be higher with the VAS-A than with the VAS-N. We also hypothesized that the QuickDASH would have a higher correlation with the PCS than with the MCS and that its correlation would be higher with the PF, RP, BP, and GH than with the VT, SF, RE, and MH subscales. The divergent validity of the QuickDASH was assessed with the correlations between the QuickDASH and age and disease duration. We hypothesized that the QuickDASH would have low or no correlation with age and disease duration.

2. Exploratory factor analysis (EFA) and Confirmatory factor analysis (CFA)

EFA was used to investigate the dimensionality of the scale to identify the underlying factor structure of the items. Half the patients (*n* = 94) were randomly selected from the whole sample of patients for EFA. Bartlett’s test of sphericity and the Kaiser–Meyer–Olkin measure of sampling adequacy were used to assess the data fit for factor analysis. High values of the Kaiser–Meyer–Olkin (greater than 0.5) and small values of Bartlett’s test of sphericity (significance level < 0.05) generally indicate that a factor analysis is useful with the data [25]. We hypothesized that the items could be extracted into one, two or more factors. All eleven items of the QuickDASH were used for the EFA. The principal axis factoring method was used for factor analysis extraction, and the direct oblimin method of oblique rotation was used for factor analysis rotation.

The fit of an a priori hypothesized structure of a scale can be tested by CFA. The patients included in the CFA were those not used for the EFA. We tested the hypothesized model after the results of the EFA for the QuickDASH were obtained. In the parameter estimates, the satisfactory fit was confirmed by a factor loading of each section higher than 0.5. For the assessment of the goodness of fit of the models, several fit indices were applied [26], including the Comparative Fit Index (CFI), Normed Fit Index (NFI), and Tucker–Lewis Index (TLI), as well as the χ^2^ divided degree of freedom (χ^2^/df) and Root Mean Square Error of Approximation (RMSEA).

##### Responsiveness

The changes in the QuickDASH scores of the participants who reported significant improvement were used for responsiveness analysis. To evaluate the responsiveness, the effect size (ES) and standardized response mean (SRM) were calculated, with the ES derived by dividing the mean of score changes by the pooled standard deviation and the SRM by dividing the mean of score changes by their standard deviation. Values greater than 0.8 indicated large ES and SRM [27].

## 3. Results

### 3.1. Participant Characteristics

The total sample size of the study was 189. All participants finished the questionnaire booklet. Thirty participants had neck pain only. The demographic data of the participants are listed in Table 1. Regarding gender distribution, women outnumbered men; the male–female ratio was about 1:1.55 for all 189 participants. The most common occupation of the patients was office workers (about 44%). The mean QuickDASH scores were 24.9 ± 22.7 (range 0–90.9) for all patients, indicating that most participants had mild to moderate disability. The mean QuickDASH scores were different between patients with neck pain only and those with neck and arm pain (14.1 ± 15.9 vs. 27.0 ± 23.2, *p* < 0.001). Sixteen participants in the chronic stage participated in the test–retest study. For the responsiveness study, about half *(n* = 30) of all the participants *(n* = 68) reported significant improvement in symptoms. The mean ages of the participants in the validity, test–retest and responsiveness studies were similar (57.9, 52.6, and 59.6 years, respectively).

### 3.2. Acceptability

For assessment of the acceptability of the QuickDASH, the entire sample of participants in the study was included. Most participants (83.6%) were found to have upper limb symptoms, and the QuickDASH had a slight floor effect (16.4%) in patients with neck pain with or without arm pain. The score range of the patients was 0–90.9; therefore, no ceiling effect (0%) was found. The QuickDASH was easy to administer and only took a short time to finish. No missing data were found (the participants answered at least 10/11 questions). Most participants answered all the questions, and only 6 (3.2%) provided no answer for one question (limitations in work or daily activities in three persons, difficulty sleeping in two persons, and recreational activities with force or impacts in one person). The score distribution of the QuickDASH scores lacked adequate normal distribution, with high skewness (1.2 ± 0.2).

### 3.3. Reliability

#### 3.3.1. Internal Consistency

Table 2 lists the results on internal consistency for Cronbach’s α, item–total score correlation, and Cronbach’s α if the item was deleted from the scale. Cronbach’s α was 0.945, indicating excellent internal consistency. The item–total score correlations were high, ranging from 0.67 (difficulty sleeping) to 0.84 (limitations in work/daily activities). Cronbach’s α with an item deleted did not increase for each question, indicating that all questions were relevant to the population with neck pain with or without arm pain.

#### 3.3.2. Test–Retest Reliability

Table 3 presents the test–retest reliability results, including both the relative reliability (ICC) and absolute reliability (SEM and MDC). The ICC value was 0.98, indicating excellent test–retest reliability. The SEM of 3.17 indicated only 3.17% of the total score range (0–100% disability). The MDC value was 8.79.

### 3.4. Validity

#### 3.4.1. Convergent and Divergent Validity

Table 4 lists the correlation coefficients of the scores of the QuickDASH and the variables used to examine the convergent and divergent validity. The convergent validity was supported by the high correlation of the QuickDASH scores with the NDI scores (|rho| = 0.80) and the BP and SF subscale scores (|rho| = 0.68 and 0.67, respectively). The QuickDASH scores were more highly correlated with the VAS-A scores (|rho| = 0.56) than with the VAS-N scores (|rho| = 0.49). The QuickDASH scores were more highly correlated with the PCS, PF, and RP scores (|rho| = 0.62, 0.54, and 0.50, respectively) than with the MCS, RE, GH, VT, and MH scores (|rho| = 0.47, 0.49, 0.41, 0.49, and 0.37, respectively). Briefly, the results showed moderate to high correlation, indicating satisfactory convergent validity. For divergent validity, the QuickDASH scores were not correlated with age or disease duration (|rho| = 0.06 and 0.096, *p* > 0.05), indicating satisfactory divergent validity.

#### 3.4.2. Exploratory Factor Analysis (EFA) and Confirmatory Factor Analysis (CFA)

EFA was used to explore the possible factor structures of the 11 items. The value of the Kaiser–Meyer–Olkin Measure of Sampling Adequacy was 0.925, and Bartlett’s test of sphericity result was significant (*p* < 0.001), indicating that factor analysis was useful with those data. Two factors had eigenvalues over 1, and two steep slopes of the scree plot confirmed the existence of two factors. The factor loadings from the pattern matrix with direct oblimin rotation also demonstrated the two-factor model for the 11 items of the QuickDASH (Table 5). The two factors were correlated, with a correlation of 0.689. Factor 1 had 8 items (Q1 to Q8) representing disability of the upper limb, named the function factor, and factor 2 had 3 items (Q9 to Q11) representing symptom information, named the symptom factor. Cross-loading was found in Q7 and Q8 (factor loadings in factor 1: 0.43 and 0.43; in factor 2: 0.51 and 0.58). Q7 and Q8 were classified in the function factor due to questions asking about interference with social activities and limitations in work or daily activities. The two-factor structure explained 80.9% of the variance. The factor loadings of the eight items (Q1 to Q8) in factor 1 ranged from 0.43 to 1.00, and the factor loadings of the three items in factor 2 (Q9 to Q11) ranged from 0.90 to 0.91.

The CFA results demonstrated that the factor loadings of the sections for the function factor and symptom factor were all satisfactory (0.83 to 0.93 for the function factor and 0.80–0.97 for the symptom factor). Several model fit indices were used for model comparison. For the two-factor model, the values of χ^2^/df (2.985) and RMSEA (0.145) were low and the values of CFI, TLI, and NFI were high (0.873, 0.805, and 0.826, respectively).

### 3.5. Responsiveness

The number of patients who participated in the responsiveness study was 68, 30 of whom reported significant improvement 3 months after the initial assessment. The average time span between the first and second assessments was 4.3 ± 1.5 months (ranging from 3 to 6 months), and the mean QuickDASH scores of the patients with significant improvement fell from 27.3 ± 17.0 to 13.0 ± 15.9. The value of the ES was 0.85 and that of the SRM, 0.82.

## 4. Discussion

The study examined various psychometric properties of the QuickDASH in patients with neck pain with or without arm pain. The results showed the QuickDASH to have satisfactory acceptability, reliability, validity, and responsiveness in patients with neck pain with or without arm pain.

### 4.1. Acceptability

For patients with neck pain with or without arm pain, the questions of the QuickDASH were easy to answer without missing values. The disadvantage of the QuickDASH was its slight floor effect (16.4%). A possible reason may be that the study included participants with neck pain whether or not they had pain in an upper limb. We found that the QuickDASH detected upper limb problems in most of our participants (83.6%), and the maximum score was 90.9. For all patients, the mean score of the NDI was 12.3 ± 10.0. When we checked the scores of the NDI against the scores of the QuickDASH of 0 (*n* = 31), the score range of the NDI was 0 to 14.45. The mean score of the QuickDASH was 24.9 ± 22.7. For the two patients with NDI scores of 0, the scores of the QuickDASH were also 0; however, for patients with NDI scores between 2 and 3 (five patients), the range of the scores of the QuickDASH was large (from 0 to 47.7). Therefore, the QuickDASH should not be used in place of the NDI. We suggest that both the QuickDASH and NDI be used concurrently to assess neck and upper limb disorders in patients with neck pain.

### 4.2. Reliability

Our study showed that the internal consistency of the QuickDASH was high (Cronbach’s alpha = 0.945), equal in quality to those in reports on patients with upper limb problems (0.92–0.94) [28,29,30,31]. The item–total score correlations were high (0.67–0.84). No deletions of single items increased Cronbach’s alpha by more than 0.1, indicating high homogeneity of all the items.

The test–retest reliability in this study was excellent. For relative reliability, the ICC was 0.98; that value was even better than those reported for patients with upper limb problems (0.92–0.94). For absolute reliability, the SEM and the MDC at the 95% confidence level were 3.17 and 8.79, respectively. An SEM value exceeding 3.17 points in group comparisons indicates an actual difference in function. An MDC value at the 95% confidence level of over 8.79 for an individual indicates that a change is real and not within the range of error of the measure. The MDC in the current study was better than those in previous reports (12.3–20.4) on patients with upper limb problems [28,32,33]. The SEM and MDC of the NDI (score range 0–50) were 3.15 and 8.74, respectively, in a study by Lue [18]. The values of the SEM and MDC of the QuickDASH were also better than those of the NDI.

### 4.3. Validity

The QuickDASH had good validity in patients with neck pain with or without arm pain, as determined by various psychometric testing methods. The hypothesis testing showed that the similar constructs were as expected, having moderate to high correlation, and the different constructs were as expected, with very low correlation. The correlation coefficient of the QuickDASH and the NDI (rho = 0.80) was high, similar to those in studies by Mehta (Pearson’s r = 0.82) and Khalifen (rho = 0.74 to 0.82) [11,13]. In See’s study, the correlation coefficient of the DASH and NDI was also high (rho = 0.77) in 24 patients with persistent whiplash-associated disorder [34]. The QuickDASH has fewer items than the DASH, and the former may be suitable for patients with whiplash-associated disorder, given its satisfactory validity. The correlation in our study was higher with arm pain (VAS-A rho = 0.56) than with neck and shoulder pain (VAS-N rho = 0.49). The correlation coefficients for PCS and the PF, RP, and BP subscales were high (absolute rho = 0.62, 0.51, 0.50, and 0.68, respectively). The QuickDASH was also highly correlated with the SF subscale (absolute rho = 0.67). A possible reason might be cross-loading in the SF subscale (factor loading in physical factor = 0.34; in mental factor = 0.39), which was previously found in a study of 7093 patients [35]. Furthermore, Q7 asks about interference with social activities.

For structural validity, we performed EFA and CFA on different samples chosen by random selection. The EFA identified two factors: the function factor (8 items, from Q1 to Q8) and the symptom factor (3 items, from Q9 to Q11). The two factors were correlated. The current study was the first to examine the structural validity of the QuickDASH in patients with neck pain with or without arm pain; furthermore, both EFA and CFA were used. For patients with carpal tunnel syndrome, the EFA also revealed a 2-factor model (Q1 to Q6: function factor; Q9 to Q11: symptom factor) [36]. The same authors, Stirling et al., also reported that the QuickDASH appeared to have a 2-factor structure in patients with Dupuytren’s disease [37]. In their study, Q7 (interference with normal social activities) and Q8 (interference with work) exhibited cross-loading, just as in our study. We have included Q7 and Q8 in the functional factor, as social activities and work are essential in daily life.

### 4.4. Responsiveness

In our study, the ES and SRM of the QuickDASH were 0.85 and 0.82 in patients with neck pain with or without arm pain. For comparison, 17 patients after carpal tunnel release for carpal tunnel syndrome were included in a responsiveness study of the Japanese version, and the ES and SRM were 0.37 and 0.54 [38]. The ES and SRM of the Korean version were 1.10 and 0.99 in patients with carpal tunnel syndrome [39]. Generally speaking, the QuickDASH has moderate to good responsiveness in patient with carpal tunnel syndrome. The QuickDASH is also used in participants with musculoskeletal conditions in the upper extremities requiring physiotherapy; the ES and SRM are reportedly 1.02 and 1.10 [32]. Different follow-up periods produced different results. For example, the ESs were 0.59, 0.78, and 0.82 at one-month, 3-month, and 6-month follow-ups in patients with upper-limb burn injuries [33].

### 4.5. Study Limitations

The limitations of our study must be noted. First, this was a single-center study. For better sampling, we included participants from multiple departments. Studies conducted at multiple centers would increase the sample size and thereby improve the generalizability. Second, the test–retest sample size was small. According to the COSMIN study design checklist suggestions (July 2019 version) [15], we have met most of the requirements for measurement error and reliability, such as using at least two measurements, ensuring patients are in stable condition, using an appropriate time interval between the two measurements, ensuring similar conditions for two measurements, and calculating the ICC, SEM, MDC and LoA. Our number of participants was inadequate; thus, performing the analysis in an adequate number of patients could further improve the quality of the results. Third, unlike previous studies, we compared the research findings for patients with upper limb problems because no studies of the psychometric properties of the QuickDASH had been conducted in patients with neck pain for comparison.

### 4.6. Clinical Implications

Over 80% of the patients had arm/shoulder/hand disorders, according to the QuickDASH. In clinical practice, we suggest that the QuickDASH could be used routinely for patients with neck pain with or without arm pain. The QuickDASH has excellent reliability, satisfactory validity, and acceptable responsiveness. The small measurement error (SEM = 3.17) and MDC95% (8.79) make this measure suitable for clinical application to check whether improvement after intervention is real in both group and individual comparisons. The QuickDASH assesses the upper limb function (functional factor) with 8 questions and the symptom (symptom factor) with three questions. We suggest that the NDI and the QuickDASH be used concurrently for patients with neck pain with or without arm pain.

## 5. Conclusions

The QuickDASH is a reliable and valid instrument for assessing function and symptoms in patients with neck/arm disorders. In the current study, the internal consistency and test–retest reliability were both shown to be excellent. In terms of absolute reliability, the SEM and MDC at the 95% confidence level were 3.17 and 8.79. The convergent and divergent validity were also good. The examination of the construct validity showed the QuickDASH to have two factors: the function factor and the symptom factor. The responsiveness was also shown to be satisfactory.

## Figures and Tables

**Table 1:** Demographic characteristics of the participants.

Characteristic Group	Validity (*n* = 189)	Test–Retest (*n* = 16)	Responsiveness (*n* = 30)
Age *	57.9 ± 11.1 (21–83)	52.6 ± 18.3 (21–75)	59.6 ± 9.8 (39–83)
Gender (male/female)	74/115	6/10	14/16
Disease duration (months) *	42.7 ± 78.0 (0.1–660)	46.4 ± 69.5 (5–270)	17.9 ± 29.3 (0.2–136)
Stage			
Acute (<6 weeks)	8.7%	0%	6.7%
Sub-acute (6 weeks to 3 months)	7.7%	0%	16.7%
Chronic (≥3 months)	83.6%	100%	76.6%
Education (%)			
Elementary school	24.3%	31.3%	23.3%
High school	50.8%	25.0%	36.7%
University	24.9%	43.7%	40.0%
Marital status (%)			
Single/widowed	23.8%	31.3%	16.7%
Married	76.2%	68.7%	83.3%
Occupation (%)			
Office worker	43.9%	50.0%	16.6%
Laborer	6.9%	6.3%	16.7%
Homemaker	22.2%	12.5%	23.3%
Unemployed	8.5%	12.5%	6.7%
Retired	18.5%	18.7%	36.7%

*: mean ± SD.

**Table 2:** Item–total score correlations of each item of the QuickDASH.

Item	Item—Total Score Correlation (r)	Cronbach’s α If Item Deleted
Q1. Opening a jar	0.73	0.940
Q2. Performing household chores	0.83	0.936
Q3. Carrying a shopping bag	0.78	0.939
Q4. Washing one’s back	0.73	0.940
Q5. Using a knife	0.78	0.940
Q6. Recreational activities with force/ impacts	0.77	0.939
Q7. Interference with social activities	0.81	0.937
Q8. Limitations in work or daily activities	0.84	0.936
Q9. Shoulder, arm, or hand pain	0.74	0.940
Q10. Tingling	0.71	0.942
Q11. Difficulty sleeping	0.67	0.943

Cronbach’s α = 0.945.

**Table 3:** Relative and absolute test–retest reliabilities.

Instrument	First Test	Retest	Bland and Altman Analysis		Relative Reliability	Absolute Reliability	
	Mean (SD)		$\bar{d}$ (SD)	LOA	ICC (95%CI)	SEM	MDC
QuickDASH (0–100)	18.1 (21.5)	19.9 (21.3)	1.4 (4.5)	−7.42–10.22	0.98 (0.94–0.99)	3.17	8.79

$\bar{d}$: mean difference in test–retest scores; LOA, limit of agreement (*d* ± 1.96 SD).

**Table 4:** Spearman’s rho correlation coefficients of the QuickDASH.

QuickDASH	NDI	VAS-N	VAS-A		
	0.80	0.49	0.56		
	PCS	MCS	PF	RP	BP
	−0.62	−0.47	−0.54	−0.50	−0.68
	GH	VT	SF	RE	MH
	−0.41	−0.49	−0.67	−0.49	−0.37
	Age	Disease duration			
	0.06	0.096			

**Table 5:** Factor loadings of the pattern matrix with direct oblimin rotation for the QuickDASH.

	Factor Loading	
Item Description	Factor 1	Factor 2
Q1. Open a jar	0.83	0.02
Q2. Performing household chores	0.84	0.11
Q3. Carry a shopping bag	1.00	−0.07
Q4. Washing one’s back	0.89	0.01
Q5. Using a knife	0.98	−0.10
Q6. Recreational activities with force/impacts	0.64	0.30
Q7. Interference with social activities	0.43	0.51
Q8. Limitation in work or daily activities	0.43	0.58
Q9. Shoulder, arm, or hand pain	0.04	0.91
Q10. Tingling	−0.03	0.91
Q11. Difficulty sleeping	−0.05	0.90

## Data Availability

The datasets generated and analyzed during the current study are available from the corresponding author upon reasonable request.

## Abbreviations

The following abbreviations are used in this manuscript:

QuickDASH	Quick Disability of the Arm, Shoulder, and Hand
NDI	Neck Disability Index
EFA	Exploratory factor analysis
CFA	Confirmatory factor analysis
